# Pressure-induced Transformations of Dense Carbonyl Sulfide to Singly Bonded Amorphous Metallic Solid

**DOI:** 10.1038/srep31594

**Published:** 2016-08-16

**Authors:** Minseob Kim, Ranga Dias, Yasuo Ohishi, Takehiro Matsuoka, Jing-Yin Chen, Choong-Shik Yoo

**Affiliations:** 1 Institute for Shock Physics, Department of Physics, and Department of Chemistry, Washington State University, Pullman, Washington 99164, USA; 2 Japan Synchrotron Radiation Research Institute, Koto 1-1-1, Sayo, Hyogo 679-5198, Japan; 3 Department of Electrical, Electronic and Computer Engineering, Gifu University 1-1 Yanagido, Gifu 501-1193, Japan

## Abstract

The application of pressure, internal or external, transforms molecular solids into non-molecular extended network solids with diverse crystal structures and electronic properties. These transformations can be understood in terms of pressure-induced electron delocalization; however, the governing mechanisms are complex because of strong lattice strains, phase metastability and path dependent phase behaviors. Here, we present the pressure-induced transformations of linear OCS (*R3m*, Phase I) to bent OCS (*Cm*, Phase II) at 9 GPa; an amorphous, one-dimensional (*1D*) polymer at 20 GPa (Phase III); and an extended *3D* network above ~35 GPa (Phase IV) that metallizes at ~105 GPa. These results underscore the significance of long-range dipole interactions in dense OCS, leading to an extended molecular alloy that can be considered a chemical intermediate of its two end members, CO_2_ and CS_2_.

A majority of materials in the universe exist under conditions of high pressure and high temperature, deep in the planets and stars. Under these conditions, first- and second-row elemental solids, usually known only as molecules or elements, form novel dense covalent or ionic solids in three-dimensional (*3D*) network structures^1^,^2^. These structures are extremely hard, have high energy density, and exhibit novel electro/optical properties. The structures and properties of these elemental solids can be viewed as nature’s “windows” to unexplored novel materials beyond the class of diamond and *c*-BN.

These transformations from molecular solids to non-molecular extended solids constitute solid-state chemical reactions occurring where compression energies are comparable to chemical bond energies, but substantially greater than those of defects and grain boundaries in solids (*i.e.*, *PΔV* *~* *ΔH*_*bond*_ *>>* *ΔH*_*defects*_)^3^. Therefore, the transition products are primarily bound by thermodynamic constraints, which can be predicted in terms of first principles in chemistry and physics^4^,^5^,^6^. However, the governing mechanisms are complex and often result in unusual phase diagrams with a large number of polymorphs, metastable phases, and path-dependent phase behaviors^7^. This complexity, commonly shared in many molecular systems at high pressures^8^, poses both theoretical and experimental challenges, particularly in understanding high-pressure behaviors of dense molecular solids in the transition regime where the long-range interactions become comparable or even greater than hydrogen bonding^9^,^10^.

Carbonyl sulfide (OCS) is the most abundant sulfur containing material in Earth’s atmosphere^11^ and is a linear triatomic molecule. It is isovalent to carbon dioxide (CO_2_) and carbon disulfide (CS_2_), which exhibit complex phase diagrams under pressure (see Fig. S1). Upon compression to 1–3 GPa, these molecules solidify into crystal structures typical of molecular solids^12^: *Pa3* for CO_2_-I^13^, *R3m* for OCS-I^14^, *Cmca* for CS_2_-I 15. The *Cmca* structure has also been observed in CO_2_-III^13^ above 10 GPa at ambient temperature and CO_2_-VII^16^ above ~8 GPa at high temperatures near the melt. However, it is generally considered that the *Cmca* phase of CO_2_-III is metastable^17^ with respect to the *P4*_*2*_*/mnm* structure of CO_2_-II^18^, in the same pressure region (10–40 GPa). Yet, it is the phase that transforms into non-molecular extended solids in both CO_2_ and CS_2_ upon further compression^1^,^19^.

The high-pressure phase behaviors of CO_2_ and CS_2_ become widely diverse as pressure increases above 10 GPa (see Fig. S1), hinting at the need for systematic high-pressure studies of OCS. CO_2_-III (*Cmca*), for example, develops distinctive strains (100 GPa/mm at ~20 GPa)^1^ and disorders in the lattice and, eventually, converts to non-molecular phases of amorphous *a*-CO_2_ near ambient temperature at 60–80 GPa^20^ or crystalline CO_2_-V upon heating (above 1500 K) at 40 GPa^1^,^21^. In contrast, CS_2_-I (*Cmca*) remains a highly crystalline molecular solid to ~10 GPa^22^, where it transforms to linear polymeric CS_2_-II (or *CS3* phase) with three-fold coordinated carbons and, then, four-folded CS_2_-III (*CS4* phase) at around 30–50 GPa and six-fold CS_2_-IV (*CS6 phase*) at 103 GPa^19^. Interestingly, these non-molecular solids exhibit very different structures and electronic properties, ranging from an optically nonlinear wide bandgap crystalline CO_2_-V to a highly disordered superconducting *CS4* phase (*Tc* = 6.7 K)^23^. As a result, their phase diagrams are intricate with an array of polymorphs exhibiting great diversity in crystal structures, chemical bonding, collective interactions, metastability, and thermal path dependent phases and phase boundaries^3^,^7^.

Despite an extensive amount of studies on CO_2_ and CS_2_, few studies have been done on OCS at high pressures^24^. This is a serious shortfall, considering the unique role of OCS as a non-centro-symmetric triatomic molecule, and underscores the significance of the present study on dense OCS to 130 GPa. Many experiments have been done in diamond anvil cells, using confocal micro-Raman spectroscopy, synchrotron x-ray diffraction, and four-probe electric resistance measurements (see Experimental Methods). The present results show a series of phase/chemical transformations in OCS that highlight the significance of long-range dipolar interactions in the transition regime of dense molecular solid and the intermediate nature of extended OCS between the two end members of extended CO_2_ and CS_2_, an important chemical concept for molecular alloys.

## Results

**Figure 1:**
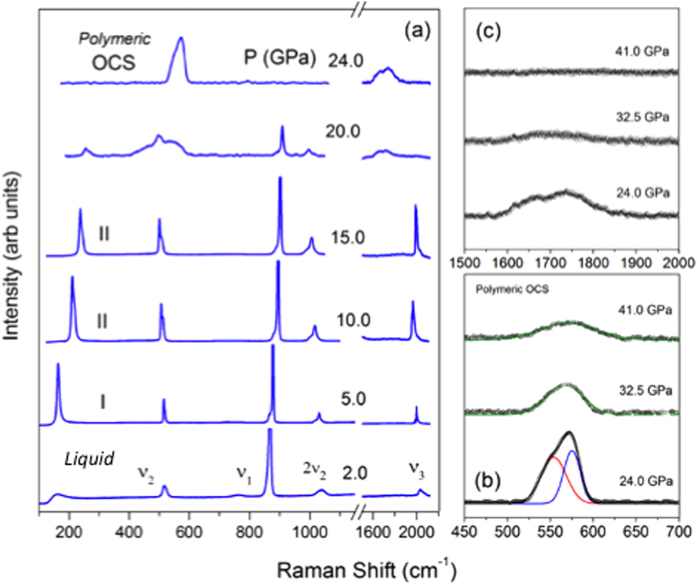
Raman spectra of OCS up to 41 GPa at room temperature. (**a**) Fundamental Raman modes, *v*_1_, *v*_2_, 2*v*_2_, *v*_3_ are denoted C-S stretching, O-C-S bending and overtone, and C-O stretching mode respectively. (**b**) bending and stretching modes of -S-(C = O)-S-, and (**c**) stretching mode of conjugated -C = O in polymeric phase.

Carbonyl sulfide crystallizes into a rhombohedral (*R3m*) structure below 134 K at ambient pressure^14^. In this structure, all OCS molecules are aligned to the body diagonal direction, representing a polar molecular crystal with an intrinsic dipole moment^25^, which is distinctive to zero momentum phases of CO_2_-I (*Pa3*) and CS_2_-I (*Cmca*). Upon compression at ambient temperature, liquid OCS solidifies into a typical molecular solid above 2.7 GPa (call it as phase I or OCS-I), as evident from the Raman spectra in Fig. 1 and the sample appearance in Fig. S2. Unlike CO_2_ and CS_2_, non-centro-symmetric OCS allows all three fundamental vibrational modes being Raman active. Those are a symmetric stretching mode *v*_1_ at 870 cm^−1^, a bending mode *v*_2_ at 517 cm^−1^, and an asymmetric stretching mode *v*_3_ at 1986 cm^−1^, at 5 GPa (Fig. 1a). In addition, the bands at 1035 cm^−1^ and 138 cm^−1^ represent the first overtone of bending mode 2*v*_2_ and a lattice mode, respectively. The relatively strong appearance of the 2*ν*_2_ is due to the Fermi resonance between the *ν*_1_ and 2*ν*_2_^24^.

**Figure 2:**
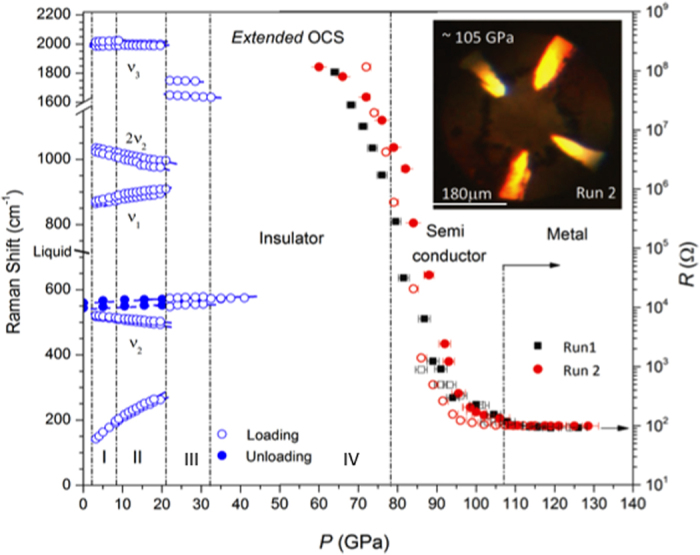
Pressure dependence of (**a**) Raman shift and (**b**) electric resistance changes of OCS. Inset shows the microscopic image of recovered OCS at 75 GPa. The black and red symbols signify, data taken during a two different runs and the closed and open symbols signify, respectively data taken during the pressure loading and unloading. The molecular phases I and II remain as crystalline solids, which polymerize to amorphous solids above 20 GPa (phases III and IV), as shown in Figs 3 and 4.

Above 10 GPa, the *v*_2_ mode increases considerably in intensity and splits into two peaks, indicating a factor group splitting associated with a structural phase transition (to a new phase, OCS-II). In addition, the overtone 2*v*_2_ at 1035 cm^−1^ develops a broad shoulder at 1023 cm^−1^ above 10 GPa. The lattice mode at 138 cm^−1^ also develops an asymmetric spectral feature reflecting the peak splitting. This lattice mode shifts rapidly with pressure at a rate of d*ν*/dP ~9.76 cm^−1^/GPa (Fig. 2).

Above 20–25 GPa, all of these sharp modes disappear and, instead, two new broad bands appear at ~547 cm^−1^ and ~1700 cm^−1^, as observed in CS_2_ when it polymerizes to a -S-(C = S)-S- configuration of CS_2_-II (or *CS3* phase) at 10 GPa^19^. As such, it is likely that these broad asymmetric peaks are, respectively, from the bending and stretching modes of -S-C-S- and the stretching modes of conjugated >C = O in an 1D polymeric configuration -S-(C = O)-S- of polymeric OCS-III. Above 32–40 GPa, the bending and stretching modes of -S-C-S- merge to form a single symmetric band centered at ~570 cm^−1^ (Fig. 1b), while the C = O stretching mode completely disappears (Fig. 1c). This indicates a further transformation to a fully saturated (or singly bonded) *3D* network solid (OCS-IV). The broad peak at ~570 cm^−1^ can then be assigned to the bending mode of the corner-sharing –O(O’)C(S’)S- tetrahedral. In fact, the observed frequency of ~570 cm^−1^ for OCS-IV is consistent with the mass-reduced bending frequencies of 700 cm^−1^ in CO_2_-V^1^ and 455 cm^−1^ in the *CS4* phase^19^. Upon further compression to 45 GPa or above, all Raman features of OCS disappear (Fig. 2), indicating a complete conversion of OCS-IV to an extended amorphous solid, similar to that in *a*-carbonia^20^. These phase/chemical changes in OCS accompany with dramatic changes in its visual appearance; OCS-I became highly polycrystalline, a pale yellow to reddish color developed in OCS-II, and dark red to black developed as OCS polymerized to OCS-III and IV (Fig. S2). The observed spectral and Raman changes are reversible at low pressures (below 15 GPa), but become irreversible above ~20 GPa. The recovered samples from 73 GPa exhibits a bright red color.

Amorphous OCS-IV is an insulator below 60 GPa, but it becomes a metal above 105 GPa, as evident from a six-order drop in electric resistance over a wide pressure range of 60 to 105 GPa (Fig. 2). The conducting phase of OCS in this pressure range is opaque and weakly reflective, as shown in Fig. 2 inset and Fig. S2. The measured resistance of the metallic phase above 105 GPa is ~100 Ω – a value typical in poor metals measured in the present DAC configuration^23^. The optical reflectivity and resistance values of poor metallic OCS are in contrast to those of good metallic CS_2_ that even superconducts at low temperatures (Fig. S3)^19^,^23^. It is also important to emphasize that the metallization in OCS-IV is *not* due to chemical decomposition, as evident from the reversible resistant change upon pressure unloading. No Raman or diffraction peak potentially associated with decomposition products such as oxygen, carbon, carbon monoxide, or sulfur – were found beyond our detection limits. Note that unlike CS_2_ 19,23 OCS-IV does not show any conductivity at the onset of polymerization (20–35 GPa), but shows only upon further compression to substantially higher pressure of 100 GPa or above. This indicates an intermediate covalence of OCS bonds between CO_2_ and CS_2_.

**Figure 3:**
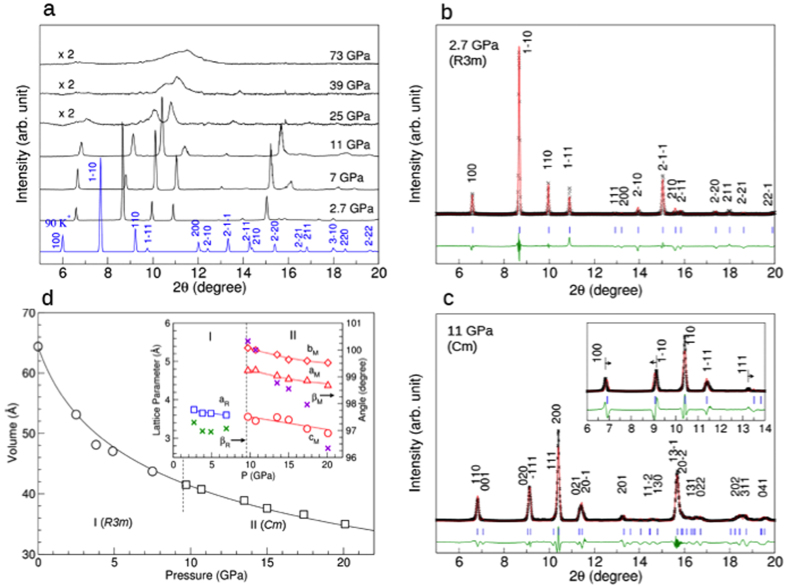
(**a**) The pressure-dependent changes of measured x-ray diffraction patterns to 73 GPa. The x-ray pattern at the bottom (in blue) is reproduced based on the crystal structure previously determined at 90 K and ambient pressure in ref. 14. (**b**) The measured (cross symbols), refined (solid red line), and difference (green line) diffraction patterns of phase I (*R3m*) at 2.7 GPa. Blue tick marks represent the Bragg reflections of the *R3m* structure. The inset shows the refined crystal structure. (**c**) The measured (cross symbols), refined (solid red line), and difference (green line) diffraction patterns of phase II (*Cm*) at 11 GPa. Blue tick marks represent the Bragg reflections of the *Cm* structure. The inset shows the refined crystal structure of phase II based on the *R3m* space group, showing a relatively poor fit. (**d**) The pressure-volume compression curve of phase I and II, showing together with the 3^rd^-order Birch-Murnaghan EOS fits. No significant volume change occurs across the phase I to II transition at ~10 GPa. The inset shows the lattice parameters of phase I and II, as a function of pressure.

The pressure-dependent x-ray diffraction changes (Figs 3 and S4) support a series of phase/chemical transformations, as seen in the Raman results. The x-ray pattern of phase I at 2.7 GPa is very similar to that reproduced by the *R3m* structure of the previous low-temperature neutron experiments (in blue, Fig. 3a)^14^.Note that phase I develops a preferred orientation on the (110) plane with pressure, becoming the most intense peak above 7 GPa. In fact, the same peak becomes the most intense peak in phase II (*i.e.,* the (111) in Fig. 3c). Therefore, the preferred orientation on the (110) plane seems to signify an incipient growth of the (111) plane of phase II as the pressure approaches transition (Fig. 3c). Note that the diffraction pattern of phase II remains that of polycrystalline OCS to ~20 GPa, above which the sharp diffraction peaks become broad or disappear. Above 40–45 GPa (Figs 3a and S5), the diffraction pattern consists of only two broad peaks (major peak at 2θ = 11.5°, minor around 19° in Fig. S5), a typical diffraction pattern for an amorphous solid.

The diffraction pattern of phase I can be readily refined with the *R3m* with Z = 1 (Fig. 3b) using the Rietveld method^26^. To improve the intensity fit, we applied a preferred orientation on the (110) plane. The refined structure gives unit cell parameters: at 2.7 GPa, a = 3.652(4) Å, α = 96.960(5)°, and ρ = 2.098(3) g/cm^3^, with reduced χ^2^ = 1.89 (Table S1). All atoms are at 1a(x, x, x) as listed in Table S1. This structure gives linear molecules aligned along the body diagonal direction, similar to that in the *Pa3* phase of CO_2_-I^13^. The calculated major bond lengths are *d*_CO_ = 1.281 Å and *d*_CS_ = 1.502 Å, representing C = O and C = S double bonds.

**Figure 4:**
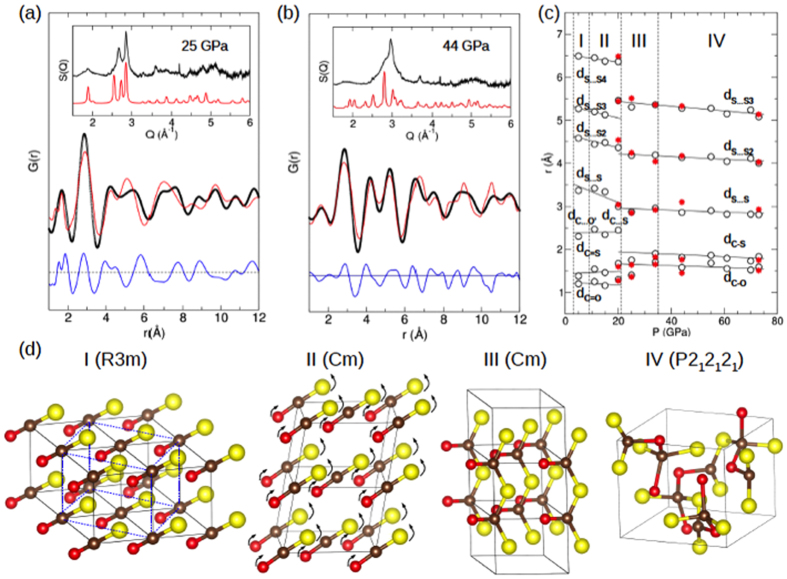
(**a**,**b**) The pressure-dependent change of measured (black circle), PDF refined (red line), and difference (blue line) G(r) patterns at 25 GPa for phase III (*Cm*) and 35 GPa for phase IV (*I2*_*1*_*2*_*1*_*2*). The insets present the measured (black) and simulated (red) x-ray scattering function S(Q) for corresponding phases. (**c**) The pressure-dependent peak shifts of the measured G(r) (open circles), plotted together with the PDF refined pair distances (red stars). Most peaks are composed by scatterings from multiple atomic pairs, however, only the dominant atomic ones are labeled. (**d**) the refined structure models for OCS polymorphs, highlighting (i) a displacive nature of the phase I to II transition mechanism, (ii) a long-range dipole interaction driven polymerization to 1D phase III, and (iii) a density-driven interlayer crossing to phase IV.

The diffraction pattern of phase II at 11 GPa (Fig. 1c) shows a slightly distorted monoclinic structure (*Cm*), which cannot be described with the *R3m* structure (the inset). For example, the *R3m* structure gives the (1–10) peak as the strongest. As such, the refinement with a strong preferred orientation on the (110) does not provide reliable atomic coordinates or relevant bond distances of C = O or C = S. On the other hand, the *Cm* structure fits considerably better in terms of peak positions, asymmetric peak profiles, and diffraction intensities. To refine the atomic parameters, we applied crystal models with both linear and bent OCS molecules. For both models, the bond lengths were constrained to 1.0 Å < *d*_C-O_ < 1.6 Å and 1.3 Å < *d*_C-S_ < 2.0 Å, which cover sufficient distances for both single and double CO and CS bonds. The results showed that the bent molecular model yielded a much better result with the structural parameters of a = 4.647(3) Å, b = 5.249(5) Å, c = 3.520(3) Å, and β = 100.10°(15) at 11 GPa, with reduced χ^2^ = 2.82. The refined atomic positions are: C(0.702, 0.0, 0.288), O(0.923, 0.0, 0.120), and S(0.467, 0.0, 0.548), yielding a layer structure with OCS molecules aligned along the face diagonal, analogous to that found in CO_2_-III (*Cmca*)^13^. The best-refined structure shows a small bending in OCS (<OCS = ~171.5°) and slightly elongated C = S (d_C_ = _S_ = 1.54 Å) but nearly the same C = O bond distances at 2.7 GPa. Applying the same refinement procedure to the data at 20 GPa, we find that the C = S distance increases to 1.59 Å, about ~6% larger than that at 2 GPa (~1.50 Å), while the C = O distance still remains nearly unchanged at 1.27 Å. Importantly, OCS becomes considerably more bent to <OCS = 156.4° at 20 GPa. This structure (Fig. 4d) has a nearest neighbor distance of 2.31 Å between carbon and sulfur along the [101] direction, the same direction that forms an *1D* -S-C( = O)-S- chain upon further compression.

Figure 3d shows the pressure dependent unit cell volumes and the 3^rd^ order Birch-Murnaghan equation of state (EOS) fits, together with the pressure-dependent lattice parameters in the inset, to 20 GPa. The compression data shows a gradual volume change across the phase I to II transition without any apparent discontinuity. In fact, these data can be fit well using a single EOS model at B_o_ = 8.75(0.85) GPa and B_o_’ = 4.20(0.29).

To investigate the local structure of disordered phases above 20 GPa, we have performed the pair distribution function (PDF) analysis (see Fig. S5)^27^. Figure 4 plots the resulting G(r) and S(Q) for (a) phase III at 25 GPa and (b) phase IV at 44 GPa. Because of the similar molecular arrangement between phase II and III, we use the *Cm* structure to fit the G(r) at 25 GPa. The refined local structure parameters and resulting bond distances reproduce the measured S(Q) (Fig. 4a inset) and G(r) (Fig. 4a), with a = 4.270 Å, b = 4.751 Å, c = 2.894 Å and β = 96.36° with ρ = 3.42 g/cm^3^. The calculated G(r) peaks at 1.37 Å, 1.72 Å and 2.8 Å, respectively, correspond to the distances for C = O, C-S, and S-S bonds, suggesting a *1D* polymer chain structure of -S-(C = O)-S- in the ac-plane with three-folded carbon atoms (see Fig. 4d). For comparison, we performed the same PDF analysis at 20 GPa (Fig. S6a) and obtained results consistent with those from the Rietveld refinement of phase II (Table S1).

For phase IV at 44 GPa we applied the structure models for both β-cristobalite (*I–42d*)^21^ and tridymite (*P2*_*1*_*2*_*1*_*2*_*1*_)^28^, both observed in CO_2_-V and CS_2_-IV. To allow the asymmetric distribution of O, C, and S atoms in the *I–42d*, we used its subgroup *I2*_*1*_*2*_*1*_*2*_*1*_. In general, the refined PDF fits are better to the tridymite structure (Fig. 4b) than to the β-cristobalite (Fig. S6b). The resulting parameters for both structures are summarized in Table S2; for tridymite a = 6.528 Å, b = 5.425 Å and c = 6.158 Å with ρ = 3.66 g/cm^3^ at 44 GPa. The estimated pair distances are d_C-O_ = 1.45 Å, d_C-S_ = 1.76 Å, d_S-S_ = 3.13 Å, and d_S-S2_ = 4.17 Å, which agree well with the measured G(r) peaks (red stars in Fig. 4c). The calculated S(Q) also reproduces the major features of the measured diffraction data (Fig. 4b inset).

Note that the PDF analysis results in a relatively low density, ρ = 3.66 (or 3.79) g/cm^3^ at 44 (or 73) GPa, compared to ~3.8 g/cm^3^ of CO_2_-V^21^ and ~4.4 g/cm^3^ of CS_2_-IV^19^ at ~70 GPa. Nevertheless, this can be understood in terms of the highly disordered structure of OCS with three- and four-fold coordinated carbons (Fig. 4d). For example, using the G(r) and calculated density, we calculated a coordination number, 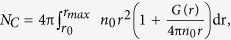 where, *r*_*0*_ and *r*_*max*_ are the beginning and the ending points of the first peak of radial distribution function, *n*_*0*_ is an atomic number density. We obtained the *N*_*C*_ values at 3.16 at 25 GPa for phase III and 3.55 (or 4.12) at 44 (or 73) GPa for phase IV, consistent with the structural model.

Figure 4c presents the atomic pair distances obtained from the Rietveld refinement below 20 GPa and the G(r) above 20 GPa, as a function of pressure. For comparison, the PDF refined G(r) are also plotted in red stars. Note that the majority of G(r) peaks at small r (especially below 2 Å) represent a combined contribution of multiple atomic pairs, whereas the peaks at large r are mainly from sulfur pairs due to the larger scattering cross section of sulfur. It is apparent then, that C = O and C = S double bonds transform to C-O and C-S single bonds in the pressure range of 20–35 GPa. Simultaneously, the second peak centered at ~2.4 Å (noted as d_C…O_ and d_C…S_) merges with the first peak at ~1.8 Å to form additional C-O and C-S bonds. The intermolecular S-S distance reduces to ~2.8 Å – approximately the S…S distance in an O(O’)C(S’)S tetrahedron. Above 35 GPa, all atomic pair distances slowly decrease with increasing pressure, signifying a densification without any major modification in the tetrahedral units. This is also apparent from the ratio of the first and second G(r) peaks corresponding to the radius-to-edge ratio of a tetrahedron, which remains nearly unchanged 1.60 at 34 GPa and 1.65 at 74 GPa for a nearly perfect tetrahedron^29^. For comparison, the same ratio for phase II is 1.81 at 15 GPa and 2.19 at 20 GPa. Thus, the results support the finding that phase IV is primarily made of singly bonded corner-sharing tetrahedral in a *3D* network structure.

## Discussion

The present structural models of OCS polymorphs provide insight into observed transition mechanisms (Fig. 4d). Note that OCS molecules are aligned to the body diagonal in phase I, whereas they are aligned to the face diagonal in phase II in a similar unit cell. At 11 GPa, two diagonal directions of the *ab*-plane, **a** **·** **b** = 4.62 Å, **a** **·** **−b** = 5.12 Å, and the c-axis, c = 3.45 Å, of phase I (*R3m*) are *very* close to **a** = 4.73 Å, **b** = 5.21 Å and **c** = 3.40 Å of phase II (*Cm*). Thus, the phase I-to-II transformation involves only a simple rotation of OCS molecules from the body diagonal to the face diagonal without a major reconstruction of the unit cell. As it transforms, the unit cell (*R3m*) becomes slightly distorted (to *Cm*), and OCS molecules become bent. The degree of bending (or <OCS angle) increases (or decreases) as pressure increases. This weakens C = S double bonds and increases the C = S bond distance. Interestingly, OCS molecules in the *Cm* phase are aligned in parallel, maximizing the dipole interaction between neighboring molecules in the [101] direction. This is contrary to the staggered arrangement of CO_2_-III in the *Cmca*^13^. Therefore, the OCS structure seems to signify the long-range dipole interaction in this dense molecular solid (phase II) pressure regime, which can be considered a driving force for the polymerization.

We consider the origin of molecular bending in phase II in terms of an orbital distortion of the highest occupied 2 π molecular orbitals (HOMO), which are doubly degenerated and fully occupied. With increased molecular bending, the HOMO orbitals split in two (2 π_xz_ and 2 π_yz_), rapidly lowering the energy of 2 π_xz_ while slightly increasing that of 2 π_yz_. Similarly, the lowest unoccupied 3 π molecular orbitals (LUMO) also split in two, again rapidly lowering 3 π_xz_ and slightly increasing 3 π_yz_. Such orbital distortion has two consequences: (i) stabilizing the bending configuration and lowering total energy, and (ii) reducing the band gap energy between the HOMO (2 π_yz_) and LUMO (3 π_xz_) orbitals, as evident from the color development in phase II. This compares with linear CO_2_ and CS_2_ molecules in the *Cmca* phase, in which molecules are aligned roughly staggered to each other (*i.e*., destructive dipole interaction). However, a long-range dipole effect may be important to understand the stability of bent CO_2_-IV in the *P4*_*1*_*2*_*1*_*2*_*1*_ structure, where CO_2_ molecules are antiferroelectrically ordered along the c-axis^30^.

Recall that it is this layered *Cm* (or *Cmca*) structure that transforms to a non-molecular solid in all three triatomic molecular solids (CO_2_, CS_2_ and OCS). However, the three transformations follow quite different mechanisms. For example, the transformation occurs abruptly in OCS (or CS_2_)^19^ to an intermediary three-fold *1D* polymeric configuration at 20 GPa (or 10 GPa), which then slowly converts to a four-fold *3D* network polymer over a large pressure region of 25–70 GPa (30–70 GPa). In contrast, the *Cmca* phase of CO_2_ develops considerable lattice strength and disorders over a large pressure range of 10–60 GPa before transforming to a non-molecular amorphous solid (*a*-carbonia or *a*-CO_2_) with three- and four-fold carbons at ambient temperature^31^,^32^. Four-fold CO_2_-V, on the other hand, forms only above 1500 K at 40 GPa, underscoring the strong covalent C = O bonds and the large transition barrier.

The corner-sharing tetrahedral structures found in extended phases of OCS, CS_2_ and CO_2_ are essentially open structures, subjected to structural distortions upon compression. This distortion increases the polarization of the CS or CO bonds and, ultimately, leads to a close packed sulfur (or oxygen) structure with small carbon atoms filled in the interstitials. This happens in CO_2_ at 900 GPa^33^. The pathway to such an extended lattice (or alloy), however, is controlled by strong kinetics, giving rise to stable crystalline phases with complex structures as well as metastable amorphous solids as seen in silicate minerals^34^. Note that this pressure-induced ionization to an extended molecular alloy differs from chemical decomposition^35^, or phase separation, to heterogeneous mixtures of sulfur (or oxygen) and carbon phases *via* thermal diffusions typically at high temperatures. Inherently, CO_2_ with its strong covalent CO bonds is more difficult to deform than OCS or CS_2_, which have more ionic CS bonds. Supporting evidence is found in the fact that the four-fold extended OCS and CS_2_ phases become completely amorphous solids above ~45 GPa, whereas CO_2_-V transforms to amorphous solid in the pressure region of 150–200 GPa^36^.

OCS-IV metallizes at 105 GPa– substantially higher pressure than CS_2_-III (50 GPa). CO_2_-V remains as a wide bandgap insulator to at least 220 GPa^36^. The calculated band structure based on the refined structure at 73 GPa (ρ = 3.8 g/cm^3^ for tridymite) shows that the band gap closes by band overlap in the Fermi level (see Fig. S7). On the other hand, the modified cristobalite structure has a small band gap (0.428 eV) that collapse beyond 120 GPa at an estimated density of 4.02 g/cm^3^. The partial density of state shows that the pressure-induced broadening of S 3p is responsible for the metallization, as found in CS_2_. Yet, the S 3p is hybridized with O 2p in OCS, which makes the gap substantially larger than in CS_2_, but smaller than in CO_2_.

Unlike CS_2_^19^,^23^ dense OCS does not show any conductivity at the onset of polymerization (20–35 GPa), but does so at a substantially higher pressure of 100 GPa and higher. This indicates an intermediate covalence in carbon tetrahedral bonds of OCS between those of CO_2_ and CS_2_. This, in turn, results in intermediate chemical behaviors of extended OCS between its two end members (CS_2_ and CO_2_). On the other hand, it is interesting to note that four-fold CS_2_-III further transforms to six-fold CS_2_-IV at ~103 GPa^23^ – the point at which OCS metallizes. While the structure of metallic OCS is unknown, the extrapolation of the present PDF results indicate a further increase in the nearest coordination number of 4.12 at 73 GPa (ρ = 3.8 g/cm^3^) to 4.66 at 100 GPa (ρ = 3.98 g/cm^3^), yet still remaining well within the regime of four-fold coordinated carbon atoms.

## Experimental Methods

Liquid OCS (99.99%, Sigma-Aldrich) was loaded at around −60 °C into a small area enclosed by a Teflon ring (~10 cm in diameter) surrounding two diamond anvils and a diamond anvil cell gasket. Membrane-driven diamond anvil cells were used with 1/3 carat, type Ia diamond anvils with 300 (or 180) μm culet, depending on the maximum desired pressure. A 200 μm-thick rhenium gasket was pre-indented to 30 μm and a 130 (or 100) μm hole was electro-spark drilled at the center of the gasket. A few small particles of ruby balls were used to determine the sample pressure by the R1 luminescence^37^.

Raman spectra for OCS were obtained using a home-built confocal micro-Raman system based on a Nd:YLF laser (used the second harmonic at 527 nm). The electrical resistances of OCS were measured at room temperature using a four-probe method^23^. A rhenium gasket was insulated using a fine powder of alumina mixed with epoxy. Five-μm thick copper electrodes were used with a 30 μm average separation distance between opposite electrodes.

Angle-dispersive x-ray diffraction experiments were performed at a dedicated high-pressure synchrotron beamline BL10XU at the SPring-8 in Japan. We used an intense, highly collimated (0.04 × 0.04 mm^2^) monochromatic x-ray (λ = 0.4134 Å) coupled with a *2D* image plate x-ray detector placed about 450 mm from the sample to obtain high-resolution diffraction images of concentric Debye-Scherrer’s rings from the sample. The image plate distance was determined based on the diffraction pattern of a powder x-ray standard, cubic CeO_2_ (a = 5.4116 Å)^38^. These diffraction images were then integrated as a function of 2θ to produce conventional one-dimensional diffraction profile using the Fit2D program^39^. Crystal structures of OCS phases were determined using the Rietveld methods with GSAS + EXPGUI^40^. To investigate the structure of amorphous phases, a pair distribution function (PDF) analysis was performed using PDFGetX3 41 and PDFGui^42^.

## Additional Information

**How to cite this article**: Kim, M. *et al*. Pressure-induced Transformations of Dense Carbonyl Sulfide to Singly Bonded Amorphous Metallic Solid. *Sci. Rep.* **6**, 31594; doi: 10.1038/srep31594 (2016).

## Supplementary Material

Supplementary Information
