# Supplementary material for: Pressure-induced Transformations of Dense Carbonyl Sulfide to Singly Bonded Amorphous Metallic Solid
Source: Sci Rep. 2016 Aug 16;6:31594. doi: 10.1038/srep31594 (PMC4985701; doi:10.1038/srep31594)
Supplement: Supplementary Information [file srep31594-s1.pdf]

Supplementary Information for

Pressure-induced Transformations of Dense Carbonyl Sulfide to Singly Bonded Amorphous Metallic Solid

Minseob Kim,[1] Ranga Dias,[1] Yasuo Ohishi,[2] Takehiro Matsuoka,[3] Jing-Yin Chen[1] and Choong-Shik Yoo,[1]

Supplementary Tables

Table S1. The crystal structural parameters of OCS-I (*R3m*) at 2.7 GPa and OCS-II (*Cm*) at 11 and 20 GPa, obtained by the Rietveld refinement of the measured x-ray diffraction in Fig. 3. The low-temperature structural parameters of OCS-I (*R3m*) at ambient pressure are also reproduced from Ref. S1 for comparison.

	OCS-I	OCS-I	OCS-II	OCS-II
Pressure	Ambient, 90K[S1]	2.7 GPa	11 GPa	20 GPa
Space group	*R3m* (Z=1)	*R3m* (Z=1)	*Cm* (Z=2)	*Cm* (Z=2)
a (Å)	4.063	3.652	4.647	4.355
b (Å)	4.063	3.652	5.249	4.801
c (Å)	4.063	3.652	3.520	3.206
β	98.80°	96.96°	100.10°	96.34°
V ($Å^3$)	64.43	47.55	84.53	66.508
ρ (g/cm^3)	1.548	2.098	2.360	3.000
O	(0.100,0.100,0.100)	(0.075,0.075,0.075)	(0.923,0,0.120)	(0.963,0,0.107)
C	(0.305,0.305,0.305)	(0.308,0.308,0.308)	(0.702,0,0.288)	(0.693,0,0.910)
S	(0.560,0.560,0.560)	(0.581,0.581,0.581)	(0.467,0,0.548)	(0.441,0,0.510)
d_{C-O} (Å)	1.202	1.281	1.272	1.273
d_{C-S1} (Å)	1.495	1.502	1.543	1.591
d_{C-S2} (Å)	3.592	3.183	2.974	2.314
d_{S1-S2} (Å)	4.063	3.653	3.505	3.201
O-C-S1	180.0°	180.0°	171.5°	156.4°

Table S2. The PDF analysis results of OCS-II at 20 GPa and OCS-IV at 44 and 74 GPa. The structure of OCS-IV was refined with tridymite ($P2_12_12_1$) and modified β-cristobalite ($I2_12_12_1$) models.

	OCS-II	OCS-IV			
Pressure	20 GPa	44 GPa	44 GPa	73 GPa	73 GPa
Space group	*R3m* (Z=1)	$I2_12_12_1$ (Z=4)	$P2_12_12_1$ (Z=8)	$I2_12_12_1$ (Z=4)	$P2_12_12_1$ (Z=8)
a (Å)	4.355	3.999	6.528	3.890	6.525
b (Å)	4.801	4.169	5.425	4.030	5.455
c (Å)	3.201	6.532	6.158	6.370	5.928
β (º)	96.33	90.0	90.0	90.0	90.0
V (Å^3)	66.52	108.9	218.08	110.25	211.02
ρ (g/cm^3)	2.999	3.664	3.660	3.996	3.782
C	(2a) (0.145,0.0,0.781)	(4c) (0.0,0.25,0.125)	(4a) (0.876,0.755,0.751) (4a) (0.602,0.542,0.920)	(4a) (0.0,0.250,0.125)	(4a) (0.870,0.754,0.753) (4a) (0.603,0.542,0.950)
S	(2a) (0.459,0.0,0.521)	(4a) (0.733,0.0,0.25)	(4a) (0.504,0.314,0.506) (4a) (0.975,0.212,0.990)	(4a) (0.726,0.0,0.250)	(4a) (0.496,0.314,0.506) (4a) (0.971,0.212,0.987)
O	(2a) (0.963,0.0,0.107)	(4b) (0.25,0.970,0.0)	(4a) (0.802,0.0,0.827) (4a) (0.726,0.557,0.727)	(4a) (0.250,0.036,0.0)	(4a) (0.802,0.0,0.807) (4a) (0.726,0.555,0.727)
d_{C-O} (Å)	1.308	1.70	1.452	1.53	1.490
d_{C-S1} (Å)	1.678	1.74	1.761	1.67	1.702
d_{C-S2} (Å)	2.638	2.82	2.85	2.70	2.79
d_{S1-S2} (Å)	3.241	2.985	3.13	3.05	2.94
O-C-S1	160.74º	103.03º	105.2º	104.60º	106.1º

Supplementary Figures and Captions:

Figure S1. **(a)** Phase and chemical transformation diagram of carbon dioxide reproduced from Ref. 7, showing a series of pressure-induced phase transitions from molecular phase I (*Pa3*) to phase III (*Cmca*) at 11 GPa and to extended amorphous-CO_2 (a-carbonia) above 60 GPa, at ambient temperature. Upon heating at pressures between 20 and 40 GPa, phase III transforms to phase II ($P4_2/mnm$ – isostructural to stishovite) and phase IV ($P4_12_12$ – isostructural to α-cristobalite). Upon laser heating at 35 - 50 GPa, phase III transforms to extended CO_2-V with four-fold carbon atoms in tridymite-like structure ($P2_12_12_1$) or β-cristobalite structure (*I-42d*), depending on the pressure and thermal paths. In the phase diagram, the letters *a*, *c* and *i* represent amorphous-CO_2 (*a*-carbonia), coesite-like CO_2 (*c*-CO_2), and extended ionic CO_2 (*i*-CO_2). The arrows signify the thermal pathways measured each phase boundaries in various measurements. The ***c̲*** notes the path-dependent nature of *c*-CO_2, produced by laser-heating of quenched phase VI. Strongly path-dependent phase IV and II boundaries underscore the intermediary nature of these phases. The *R-3c* structure was observed from the quenched single crystal phase IV at ambient temperature and 15 GPa, which was originally grown in the stability field of phase VI at 11.7 GPa and 830 K [see Ref 16].

(b) The phase diagram of carbon disulfide reproduced from Ref. 23, showing a series of pressure-induced transitions from a transparent molecular solid phase I (*Cmca*) at 2 GPa, to a black polymer phase II (or *CS3 phase*) of $(\text{-S-(C=S)-})_p$ with three-folded carbon atoms bonded to sulfur atoms at 10 GPa and then to a highly reflective polymer phase III (or *CS4 phase*) with four-folded carbons above 40-50 GPa. The *CS4* phase becomes

magnetically ordered below ~42K and even superconducting at ~6.2K, both observed over a large pressure range from 50 to 100 GPa. Above 100 GPa, four-fold CS4 phase further transforms to phase IV (or CS6 phase) with six-fold carbons, which behaves like correlated intermetallic "molecular" alloy.

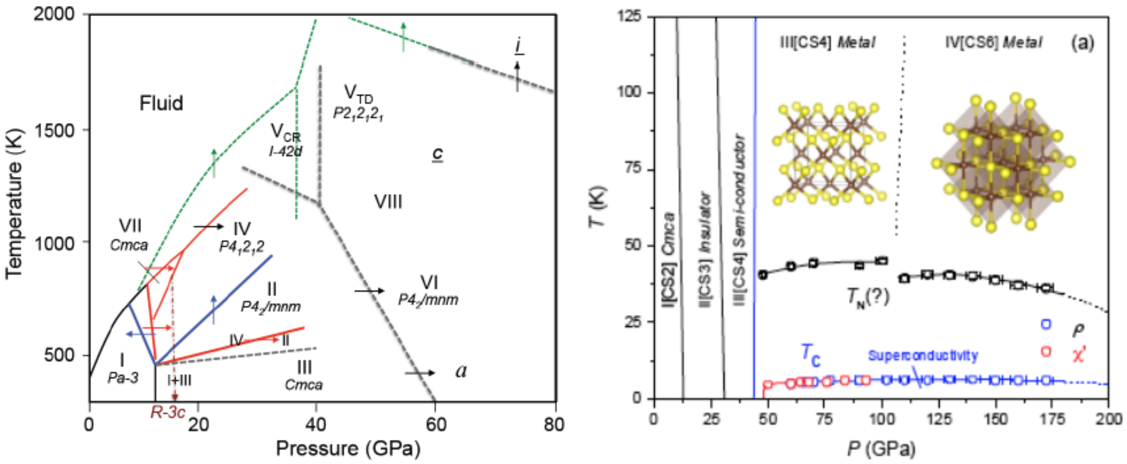

Figure S2. The microphotographs of OCS samples, showing the pressure induced visual appearance change from a transparent fluid (a), to a molecular solid (*R3m*) at ~ 2.8 GPa (b), then to a black polymer above 22 GPa $(\text{-S-(C=O)-})_p$ with three-folded carbon atoms (c, d), and eventually to a reflecting extended solid above 100 GPa at ambient temperature (e). The recovered sample from ~75 GPa (f) shows the irreversible red polymeric OCS at ambient temperature, as previously noted above 17 GPa.[S2]

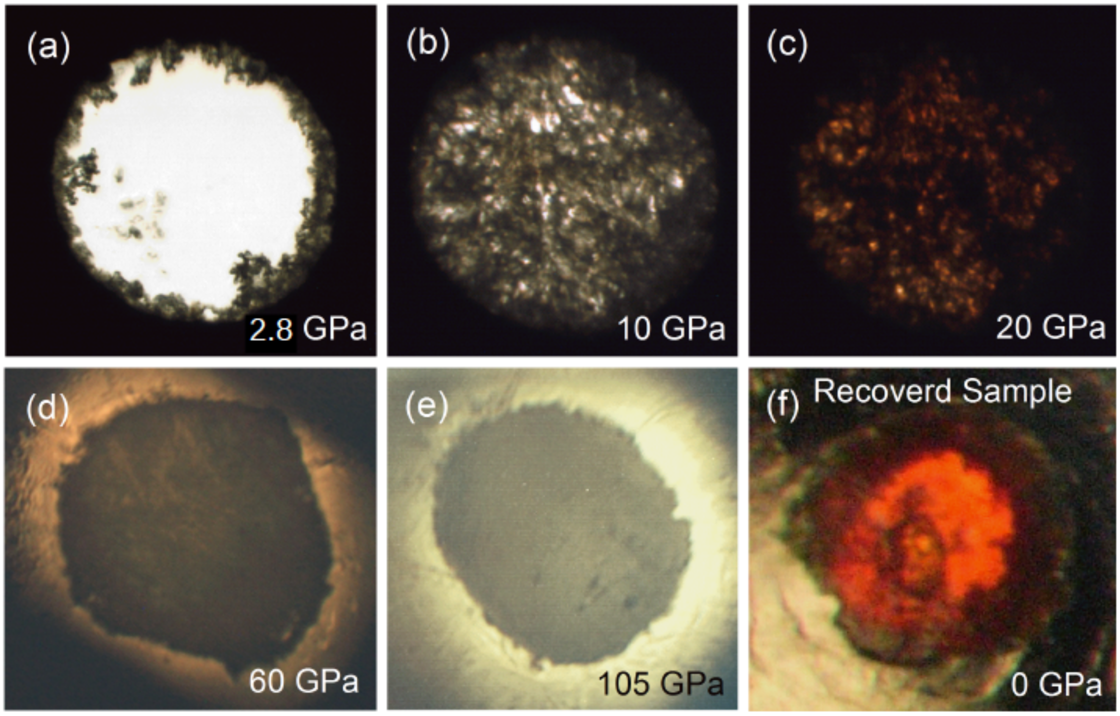

Figure S3. The pressure-induced resistance changes of OCS and CS_2, showing the insulator to metal transitions in comparison. In the inset the photographic images of metallic OCS at 105 GPa and CS_2 at 55 GPa. The plot shows substantially higher optical reflectance and lower resistivity in metallic CS_2. The date for CS_2 was reproduced from the previous work.[S3]

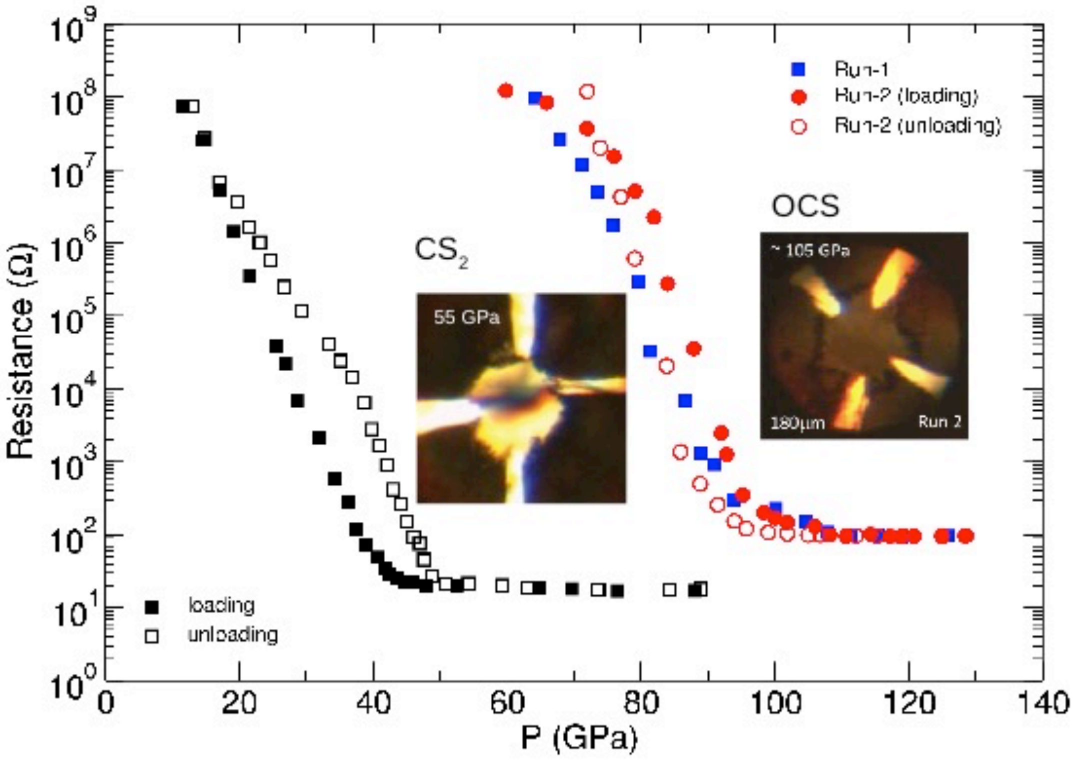

Figure S4. Pressure dependent changes of the d-spacings of OCS polymorphs to 80 GPa, showing the onset pressures of phase/chemical transformations: from phase I (*R3m*) to II (*Cm*) at ~10 GPa, III (*disordered 1D polymer*) at 20 GPa and IV (*disordered 3D network polymer*) at ~35 GPa. The d-spacings at ambient pressure (blue squares) are from the previous low-temperature neutron measurements at 90 K.[S1] The phase I-to-II transition is evident from the distinctive slop change of the pressure-dependence d-spacing shifts at 10 GPa. The transformations of crystalline phase II to disordered polymeric solids III and IV are evident by the disappearance of sharp diffraction lines and the emergence of broad diffraction bands at 20 and 35 GPa, respectively.

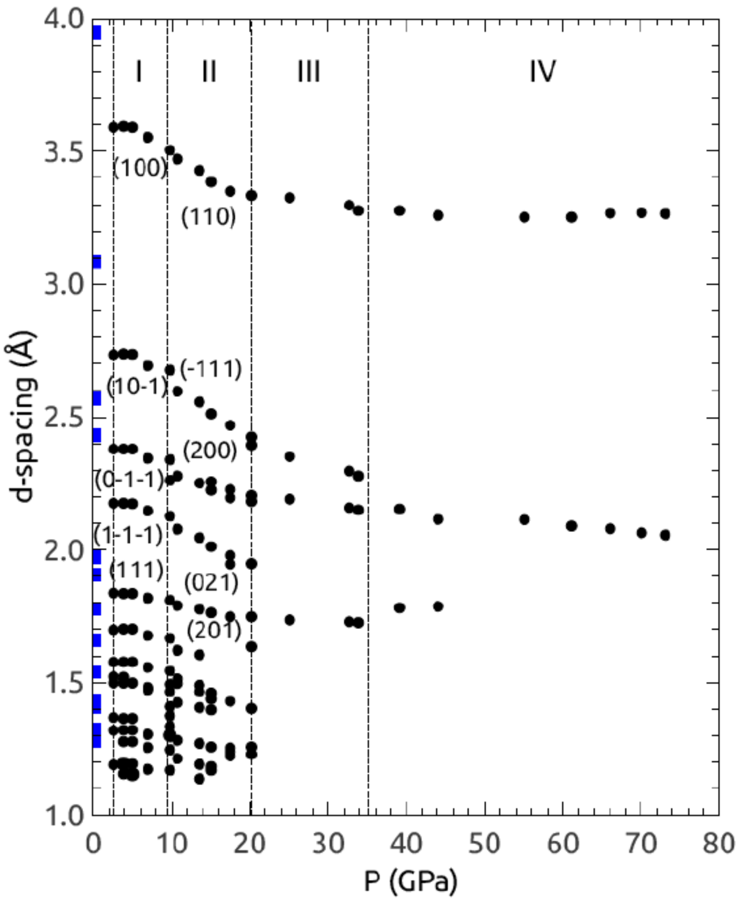

Figure S5. (**a**) X-ray diffraction patterns of OCS measured as increasing pressures at room temperature. The sharp diffraction peaks at low pressures become weak and disappear as pressure increases. The diffraction patterns above 25 GPa are those of highly disordered solids, consisting only of several broad bands.

In order to analyze the structure of disordered phases, the basic background diffraction pattern was obtained from the 5 GPa diffraction data, after subtracting the sharp diffraction lines and correcting a small pressure dependent x-ray scattering change by a linear function (mainly due to the Compton scattering and the geometric effect of DAC). The dotted blue line at 73 GPa, for example, represents the background, and the red diffraction curve at the bottom represents the background subtracted diffraction pattern.

(**b**) The pressure-dependent change of the total scattering function S(Q) as plotted in Q. The S(Q) was obtained from the background subtracted x-ray diffraction patterns, using PDFGetX3. The S(Q) patterns below 20 GPa consist of sharp peaks patterns, whereas those above 25 GPa consist of a few broad features.

(**c**) Pressure dependence change of the G(r) obtained by Fourier Transformation of the measured S(Q) in Fig. S3b:

$$G(r) = \frac{2}{\pi}\int_{Q_{\min}}^{Q_{\max}} Q[S(Q) - 1]\sin(\mathrm{Qr})\,\mathrm{dQ},$$

where $Q = 4\pi/\lambda \sin(\theta)$ and Q_{max}=6.3 $Å^{-1}$ was applied. The results show a significant change of the G(r) between 15 GPa and 25 GPa, associated with the polymerization of phase II. The board band centered at ~3.1Å at 20 GPa represents a convolution of the

second and third peaks of phase II at 15 GPa.

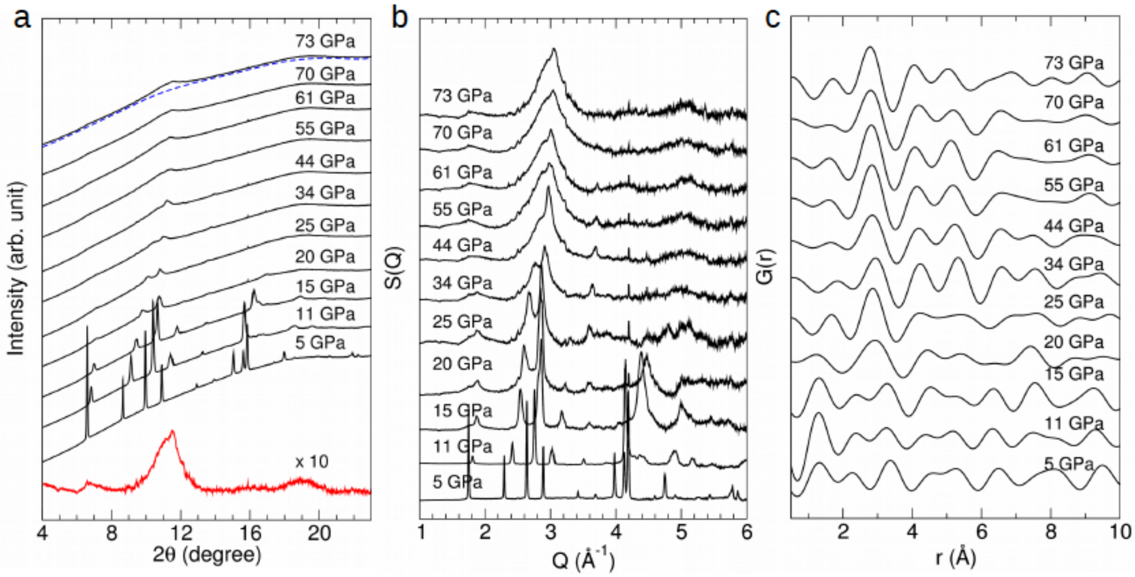

Figure S6. (**a**) The measured (black circle), PDF refined (red line), and difference (blue line) G(r) patterns at 20 GPa, based on the *Cm* structure (left-inset). The right-inset presents the measured (black) and simulated (red) x-ray scattering function S(Q) for corresponding phases. The resulting structure shows a molecular nature of OCS, in agreement with the refined molecular crystal structure for phase II.

(**b**) The measured (black circle), PDF refined (red line), and difference (blue line) G(r) patterns at 44 GPa based on the β-cristobalite structure model (left-inset). The right-inset present the measured (black) and simulated (red) x-ray scattering function S(Q) for corresponding phases. Clearly, the calculated fits to measured S(Q) and G(r) are not as good as those obtained by the tridymite model (Fig. 4b).

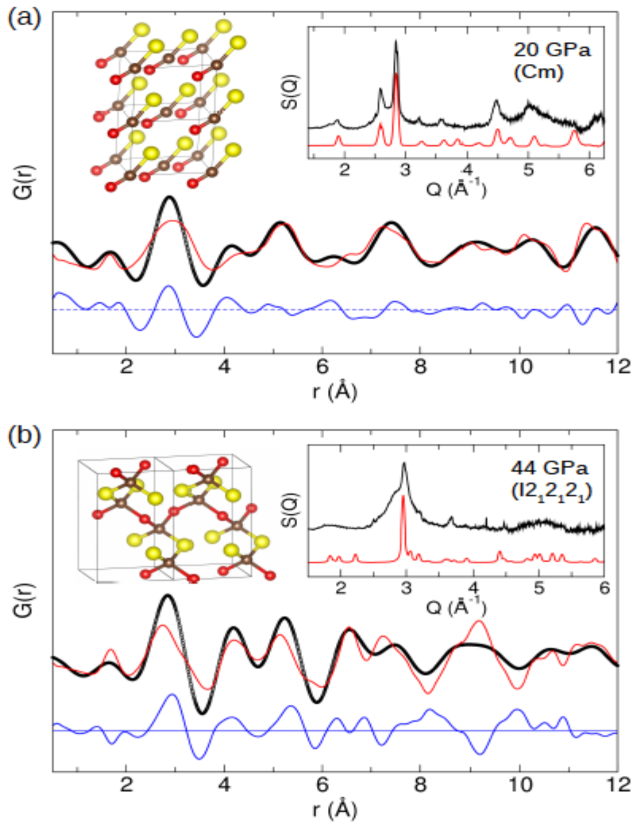

Figure S7. The electronic band structures and the projected density of states of metallic OCS computed for modified cristobalite (*$I2_12_12_1$*) at 120 GPa and tridymite (*$P2_12_12_1$*) structures at 73 GPa, using the first principles full-potential linearized augmented-plane wave (FP-LAPW) code,[S4] employing the projected augmented wave potentials[S5] with the Perdew-Burke-Ernzerhof functional.[S6]

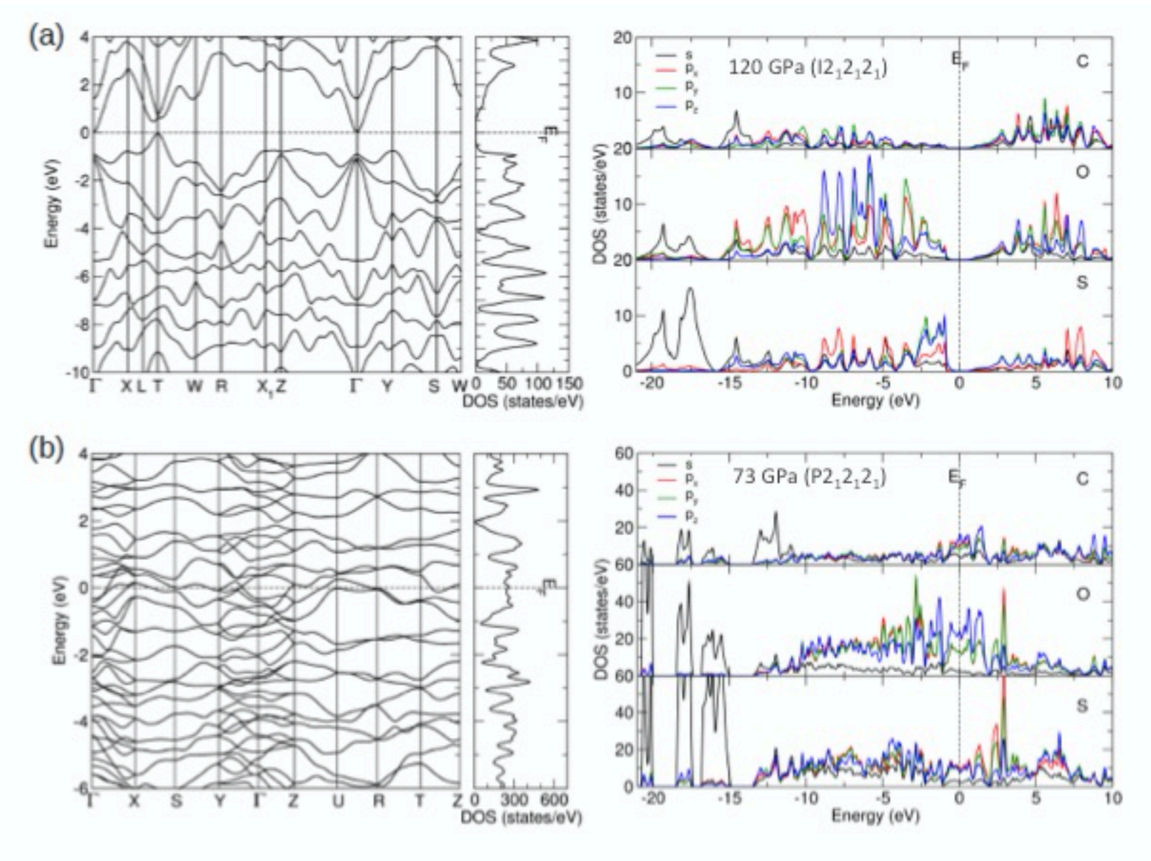

Supplementary References

S1. Overelle, J.S., Pawley, G.S. & Powell, B.M. Powder refinement of carbonyl sulphide, *Acta Cryst. B* **38**, 1121-1123 (1982).

S2. Overelle, J.S., Pawley, G.S. & Powell, B.M. Powder refinement of carbonyl sulphide, *Acta Cryst. B* **38**, 1121-1123 (1982).

S3. Dias, R.P., Yoo, C. S., Kim, M. & Tse, J. S. Insulator-metal transition of highly compressed carbon disulfide, *Phys. Rev. B*. **84**, 144104-1-6 (2011).

S4. Andersen, O.K., Formalism for the LAPW method, *Phys. Rev.* ***B*** 12, 3060-3083 (1975).

S5. Kresse, G. & Joubert D. From ultrasoft pseudopotentials to the projector augmented-wave method, *Phys. Rev. B* **59**, 1758-1775 (1999)

S6. Perdew, J.P., Burke, K. & Ernzerhof, M. Generalized gradient approximation made simple, *Phys. Rev. Lett*. **77**, 3865-3868 (1996).
